# Pharmaceutical Inspection Co-operation Scheme: An Overview

**DOI:** 10.7759/cureus.69043

**Published:** 2024-09-09

**Authors:** Prasanna Gayathri B, Kamaraj R

**Affiliations:** 1 Pharmacy, SRM Institute of Science and Technology, Chennai, IND

**Keywords:** accession, gmp compliance, gmp requirements, information exchange, inspectorates, international cooperation, networking, pharmaceutical inspection co-operation scheme (pic/s), pic/s gmp forum, pre-accession.

## Abstract

To address the challenges posed by the globalization of regulatory agencies, it is essential to harmonize regulatory requirements, assess Good Manufacturing Practice (GMP) compliance, license production facilities, manage product recalls, and improve information exchange. The Pharmaceutical Inspection Co-operation Scheme (PIC/S) provides a robust framework for tackling these global issues. Through the PIC/S GMP Forum, PIC/S facilitates informal interactions among non-member authorities, professionals, and various organizations with the Committee, fostering networking and collaboration. Furthermore, PIC/S has developed a comprehensive guide on GMP requirements for inspectorates and organizations, ensuring consistent standards across borders. The scheme collaborates with other organizations to enhance and complement collective efforts. With 56 countries involved in PIC/S, whether as members or through the Accession and Pre-Accession Process, the exchange of inspection reports among authorities verifies compliance with PIC/S standards, leading to fewer and more targeted inspections and reducing redundancy. This study's purpose is to establish uniform GMP standards by providing extensive training for inspectors and promoting collaboration and networking among regional and international bodies, regulatory authorities, and other relevant organizations. This approach seeks to build trust in inspections, optimize resource use, and foster a more efficient regulatory environment. Through these efforts, PIC/S continues to play a pivotal role in advancing global pharmaceutical regulatory practices.

## Introduction and background

The Pharmaceutical Inspection Convention (PIC) of 1970 was extended in 1995 with the creation of the Pharmaceutical Inspection Co-operation Scheme (PIC/S). It was established by “European Free Trade Association” in 1970. The organization aims to promote effective and collaborative efforts in the realm of “Good Manufacturing Practice (GMP)” with several goals. These goals include the implementation, development, and establishment of uniform GMP guidelines and processes for quality auditors in the pharmaceutical market. Furthermore, the program promotes collaboration and interaction among relevant regulatory bodies, as well as local and global groups, in order to establish confidence. All decisions within the scheme are made unanimously. Currently, there are 56 participating authorities [1]. Table 1 shows the comparison between PIC and PIC/S [2].

**Table 1:** Comparing PIC and PIC/S PIC: Pharmaceutical Inspection Convention; PIC/S: Pharmaceutical Inspection Co-operation Scheme

PIC	PIC/S
Conventional	Structure
Between Nations	Between Regulators
Official Agreements	Unofficial Agreements
Has legal standing	Has no legal standing
Concentrate solely on observation	Pay attention to formulating guidelines, training, and inspection

History behind the establishment of the PIC/S scheme

Established in 1995, PIC/S expanded the scope of the PIC which was initially established by the European Free Trade Association (EFTA) in October 1970 under the title "The Convention for the Mutual Recognition of Inspections in Respect of the Manufacture of Pharmaceutical Products." The initial members of the PIC consisted of the 10 EFTA countries at that time, with more members joining at a later stage.

The European Union (EU) introduced its own manufacturing practice guidelines in 1989, aligning them with this guideline's criteria. Since then, the EU and PIC/S guidelines have developed similarly. Initially, the European Commission was the only entity authorized to execute contracts with non-European countries, but it was not a party to the 1970 PIC. This discrepancy between European law and the PIC prevented EU member countries from negotiating with those wishing to join the PIC.

This resulted in the establishment of a less formal and more flexible scheme without legal character, aimed at fostering understanding among health authorities. Consequently, the scheme works alongside the existing system. Over time, it enhanced collaboration and consensus among health authorities and government agencies, resulting in working together under both frameworks. The creation of the system in 1995 ultimately led to the development of the current framework [3].

## Review

Mission

It is essential to establish and promote standardized GMP regulations and guidance documents to uphold high-quality industry practices. This involves providing training to competent authorities, particularly GMP inspectors, to ensure they are updated with the latest skills and knowledge. Furthermore, the regular evaluation of GMP inspectorates is vital for upholding stringent inspection standards. Promoting collaboration and networking among competent authorities and international organizations also contributes to effective implementation and adherence to these standards, creating a unified global approach to GMP practices.

The purpose of the pharmaceutical inspections and GMP are two areas in which PIC/S has been a trailblazer. The adaptability of the system to a dynamic environment, particularly with regard to globalization, has been impressive. In the entire world, it is the only organization that only handles GMP. With an emphasis on Good Manufacturing and Distribution Practice (GMDP) in the areas of training and standards, PIC/S's mandate will expand to GxP as of 2023. This aligns with PIC/S's new mission to enhance public health by spearheading the creation and execution of inspection frameworks for human and veterinary medicines by harmonizing standards and providing regulatory inspectors with top-notch training worldwide.

Vision

PIC/S takes pride in its role as a purely technical organization in regulatory GMP, maintaining a clear stance of non-political involvement and non-discrimination. As a leading "think-tank" in GMP, it offers a platform for expert debate and the development of new ideas. The foundation of PIC/S is based on consensus and mutual trust, treating all members as equals regardless of size or wealth. This principle allows for compromise and collaboration, ensuring that no member is left isolated. Mutual trust is fostered through voluntary cooperation and the requirement for equivalence assessments before membership, allowing for smooth and voluntary exchange of information. Driven by its participating regulatory authorities, PIC/S operates with flexibility and efficiency, avoiding bureaucracy and high costs. Members are actively engaged, contributing to events and organizational functions. The strength of PIC/S is evident in its informal networking and the strong personal connections among members and inspectors, which support brainstorming and the sharing of ideas [4].

Members

**Figure 1:**
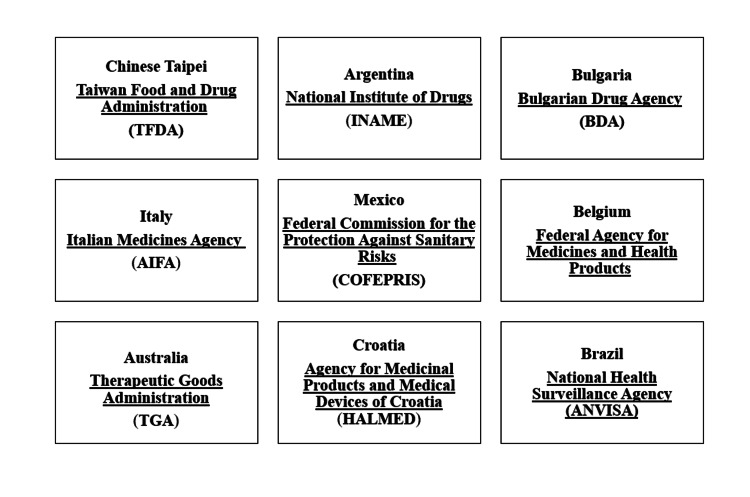
PIC/S countries and participating authorities This is an original illustration [5].

There are 56 participating authorities that are involved, and a few of them are highlighted in Figure 1 shown below [5].

Structural overview

It is a collaborative effort by regulatory agencies, designed to be flexible, dynamic, and proactive. The council, consisting of members from participating authorities, oversees the scheme's operations, with all decisions made by consensus. This council is supported by seven working groups (for example: inspector training and GMDP harmonisation), an executive bureau that gives direction for the group during conferences and an adequate secretary that provides assistance for the council, working groups, office, and participating authorities [6].

Legal framework

Since 2004, the organization has operated as a Swiss association, following the guidelines set out in Articles 60 and subsequent sections of the Swiss Civil Code. From 1971 to 2003, it had a separate legal identity with the EFTA providing the secretariat. On June 3, 2003, during a meeting in Bratislava, the decision was made to establish a Swiss association under Articles 60 and following the Swiss Civil Code. The Volunteer Red Cross International Committee (ICRC) and other globally active organisations also hold this status in Switzerland, which is not based on a treaty like UN specialized agencies. Starting January 1, 2004, the organization became self-sufficient and set up its own secretariat. As of November 11, 2004, it has been officially registered as a Swiss association with the legal name PIC/S [7].

Governance

**Figure 2:**
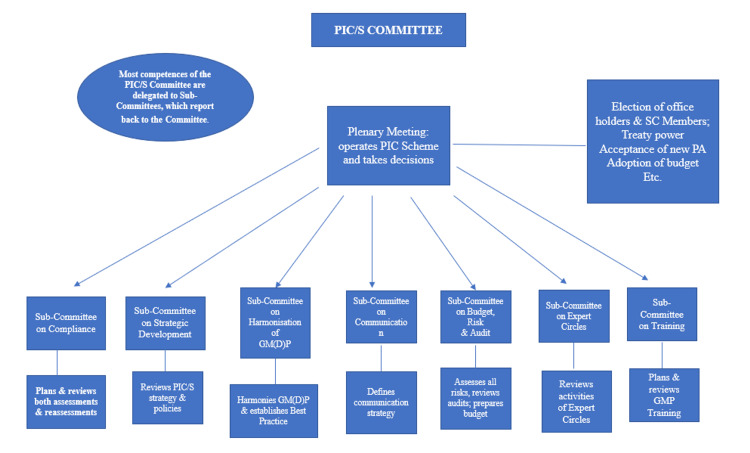
Arrangement of the sub-committees This is an original illustration [8].

The decision-making (or legislative) body consists of representatives from all participating authorities. Within this body, there are multiple working groups and seven subcommittees. The executive body includes the secretary, the seven chairs of the subcommittees, the immediate former chairperson, the deputy chairperson, and the chairperson. The secretariat provides support to both the executive body and the decision-making body in their respective roles. The arrangement of the sub-committees can be found in Figure 2 provided below [8].

Arrangement of the sub-committees

The new sub-committee structure, implemented on January 1, 2014, aims to enhance the involvement of all PIC/S participating authorities. This structure is designed to create a more efficient and collaborative environment within PIC/S, with each sub-committee responsible for its specific core areas and leading policy formulation. It also addresses the needs of PIC/S’s growing membership. The flexibility of the PIC scheme allows it to operate swiftly and proactively, with oversight provided by the PIC/S committee, composed of representatives from the participating authorities. Decisions are made through consensus. The committee is supported by seven sub-committees, each focusing on areas such as inspector training and GMDP harmonization. In addition, an executive bureau provides leadership between meetings, while a small secretariat offers assistance to the committee, sub-committees, bureau, and participating authorities in their respective tasks [8].

Committee

**Figure 3:**
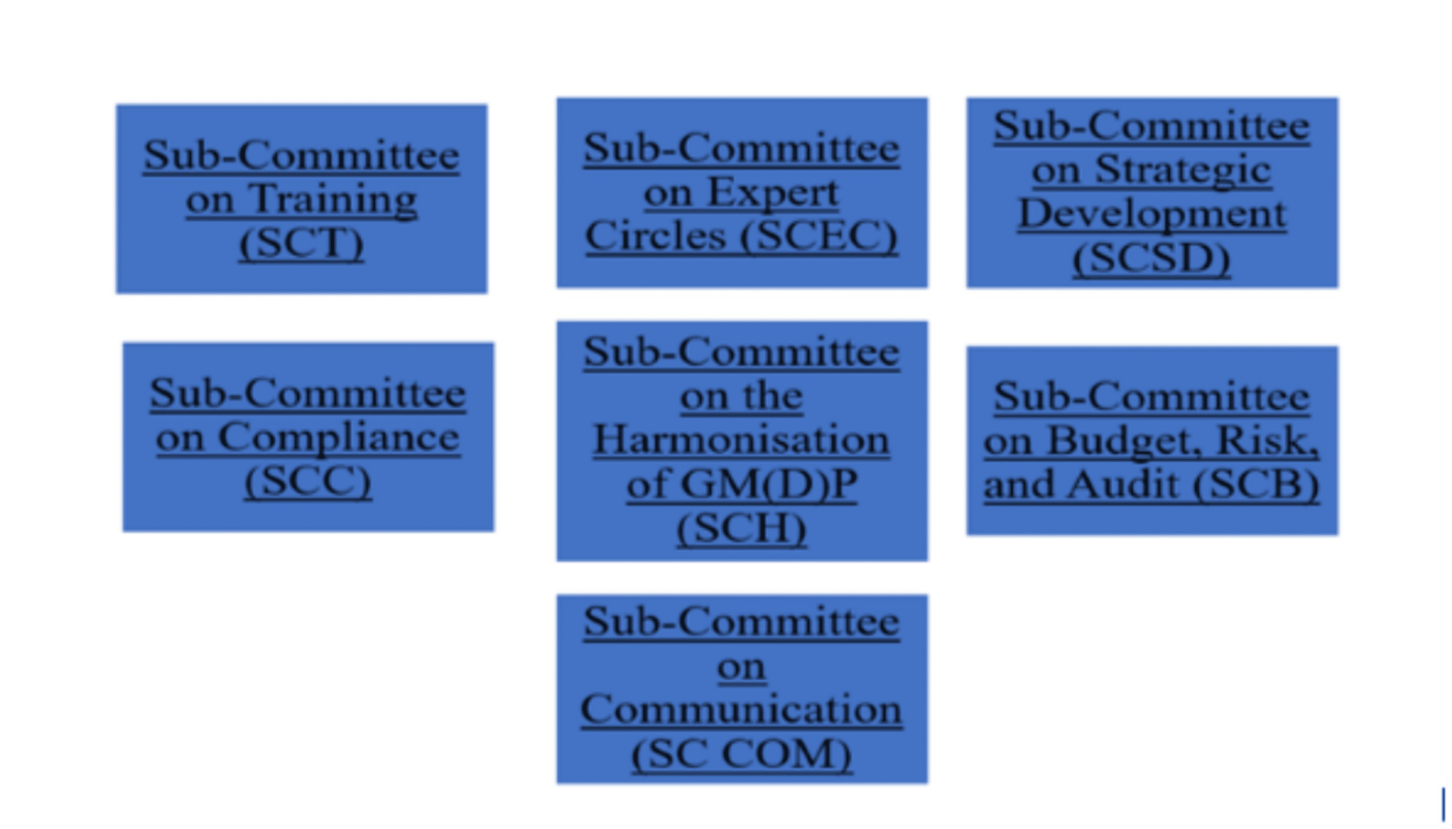
Seven sub-committees This is an original illustration [9].

The committee of officials, which is made up of members from PIC contracting states, and the participating authorities of the scheme are included. The committee is the collective term for both of them. One vote is held by each participating authority. Two authorities from the same nation may occasionally share a single vote (for example, the Federal Ministry of Health in Germany and ZLG share a single vote). Collective decision-making is used. The chairperson provides direction to the organisation as well as after meetings. At least two gatherings are convened annually to advance the objectives outlined in the PIC scheme, which also outlines the purposes of the PIC/S working groups. To promote mutual training among inspectors, methods like joint visits for harmonizing inspections and seminars on GMDP standards are essential. Financial rules should ensure transparency, with approved accounts and annual budgets supporting these efforts. An executive bureau will oversee initiatives, while negotiations and agreements help align practices across regulatory bodies. If necessary, the committee may establish sub-committees, draughting groups, working groups, or expert circles that will report back to the committee, according to the committee's rules procedure. The committee has received support in carrying out its duties from the seven sub-committees since 2014. Figure 3 below provides detailed information about the seven sub-committees [9].

Executive bureau

The PIC/S deputy chairperson, the person who served as the chairperson immediately prior, the panel, and the executive administrative assistant (who does not have voting rights) all support the PIC/S chairperson in carrying out their duties. Their combined presence makes up the executive bureau (EB). By Swiss legislation, every EB member needs to register with the Geneva Trade Registry. The PIC scheme specifies the scope of the PIC/S EB's mandate, which calls for meetings as frequently as needed and in between committee sessions.

The duties of the scheme include monitoring its operations, reviewing the annual budget by the fiscal rules, establishing strategic directions and assisting in decision-making, overseeing the secretariat and serving as an employer for its employees. The decision-making body receives reports from the EB. It typically meets in tandem with the PIC/S committee and has its own set of procedural rules [10].

Secretariat

The PIC programme stipulates that the committee will choose a secretariat to oversee the amenities and conference facilities. It might also offer secretarial services to other organizations. The secretariat is specifically responsible for (a) organizing committee meetings and (b) carrying out the decisions and suggestions made by the committee. It is composed of an accountant, a deputy secretary, an assistant secretary and a secretary to the PIC/S committee. The staff agreement, which governs their working conditions, governs the appointments of all secretariat staff members, which are made by the EB [11].

How to join PIC/S

Before an authority is approved, it undergoes a thorough evaluation to determine its ability to implement an inspection system comparable to current standards. This evaluation includes a review of the authority's inspection training, rules and regulations, quality oversight mechanisms, GMP audits, licensure processes, and other relevant factors. Following this evaluation, a delegation observes certain inspectors conducting standard GMP inspections. Achieving membership can take several years, during which the PIC/S committee may recommend adjustments and enhancements, including re-inspections if necessary, to ensure the effective implementation of remedial measures. Additionally, PIC/S conducts a gap analysis to identify areas where its criteria are not being met. As part of the collaborative reassessment process, existing participating authorities are regularly evaluated for equivalency to ensure that the same high standards are maintained by both newly appointed candidates and current members [3].

Becoming a member

A thorough evaluation is conducted before membership in order to ascertain if a regulatory body possesses the infrastructure and knowledge required to put in place an inspection system like to that of existing members. Examining the authority's quality system, regulatory obligations, inspector education, and GMP inspection and licensing processes are all part of this assessment. A group of employees then comes to see inspectors carrying out routine GMP examinations [12].

Accession procedure

**Figure 4:**
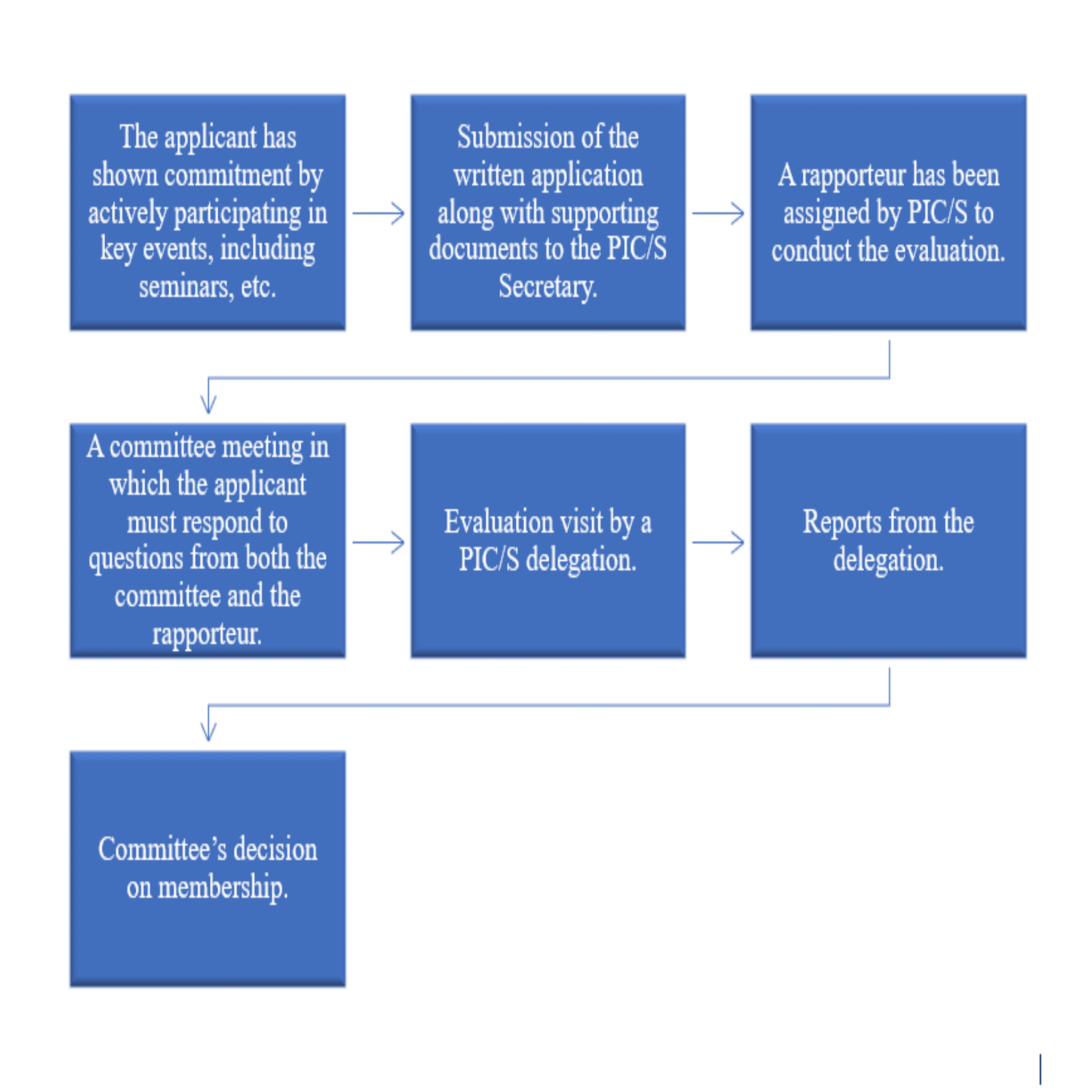
Accession procedure step-by-step This is an original illustration [13].

Accordingly, the accession procedure takes a lot of time. This is because it will require some time for you to finish your application and submit the required translations of the additional materials. Furthermore, to meet PIC/S criteria, your inspectorate might need to take action (such as improving its quality system or educating inspectors), and it also needs to provide the national industry enough time to follow the PIC/S GMP guide. Finally, the PIC/S council convenes biannually to review applications. It should be noted that there is a limit of six years to effectively finish the accession process. The step-by-step accession procedure is outlined in Figure 4 [13].

Pre-accession procedure

An optional assessment phase before the accession procedure is called the pre-accession procedure. The aim is to offer relevant governing authorities a clearer insight into PIC/S and its requirements for becoming a PA, so they can be more interested in becoming PIC/S participating authorities (PA). It has been suggested that your inspectorate use the pre-accession procedure. The individual expresses uncertainty over its compliance with PIC/S requirements, they have not established an inspectorate quality system as recommended (PI 002), and they have not adopted the GMP guide or a similar guide. Additionally, they have not consistently participated in relevant training activities.

The procedures are designed to help interested competent authorities recognize the gaps between their own GMP regulatory compliance programme and PIC/S necessities both domestically and internationally. The pre-accession procedure does not constitute consulting, nor will it include any follow-up with the remedial measures taken to close the gaps that have been found. The pre-accession process takes two years [14].

Accession requirements

GMP inspectorates with inspection systems similar to those of existing PIC/S members are qualified to become members. A law about pharmaceutical products, a GMP guide that satisfies PIC/S quality system criteria and is comparable to it, a GMP inspectorate, and skilled GMP inspectors are the primary prerequisites.

Requirements for membership

For Members

CHF 10,400 (Swiss Francs) is the annual regular membership fee. The whole membership pays for it. It should be noted that PIC/S membership entails somewhat higher dues because of regular attendance for members; it is customary to attend two PIC/S council meetings annually, one seminar each year, three expert circle meetings annually, and two to three joint visits yearly. These are all one- to three-day events that happen in different PIC/S countries across the globe. Hence, it is important to guarantee a matching travel budget.

For Applicants

A registration cost, which is equal to the annual regular membership price (CHF 10,400 for applications filed starting in 2024), must be paid by applicants. This amount is assessed when all completed applications have been sent to the PIC/S secretariat. Additionally, applicants must pay a yearly expense equal to 80% of the subscription cost that current subscribers have paid (CHF 8,600) for the duration of the assessment.

For Pre-Applicants

Applicants are not obligated to make a payment for registration. Nevertheless, they are expected to remit a yearly fee amounting to eighty percent of the enrolment cost (CHF 8,600) during the pre-assessment process, just like the applicants. External agencies interested in joining are allowed to participate in the decision-making body's meetings. They are also welcome at expert circle meetings and seminars. Participation in these events is encouraged as it offers a chance to become familiar with the organization and interact directly with current members. For example, they can use these opportunities to discuss information sharing about their GMP systems and inspection processes [15].

List of PIC/S guidelines

Guidance for Industry

Table 2 contains detailed guidelines and references for the industry [16].

**Table 2:** Industry guidance

GUIDELINE	REFERENCE
Concept Paper on the Revision of Annex 11 of the Guidelines on Good Manufacturing Practice for Medicinal Products | Computerised Systems	Concept Paper on the Revision of EU-PIC/S GMP Annex 11
Concept Paper on the Revision of Annex 4 of the Guidelines on Good Manufacturing Practice | Manufacture of Veterinary Medicinal Products other than Immunologicals	Concept Paper on the Revision of EU-PIC/S GMP Annex 4
Concept Paper on the Revision of Annex 5 of the Guidelines on Good Manufacturing Practice for Medicinal Products | Manufacture of Immunological Veterinary Medicinal Products	Concept Paper on the Revision of EU-PIC/S GMP Annex 5
Explanatory Notes for Pharmaceutical Manufacturers on the Preparation of a Site Master File (SMF)	PE 008-4
Joint PIC/S-EMA Concept Paper on the Revision of Annex 1 (Manufacture of Sterile Medicinal Products)	PS W 01 2015
PIC/S GMP Guide (PE 009-17) Part II	PE 009-17 (Part II)
PIC/S GMP Guide (PE 009-17) Annexes	PE 009-17 (Annexes)
PIC/S GMP Guide (PE 009-17) Introduction	PE 009-17 (Intro)
PIC/S GMP Guide (PE 009-17) Part I	PE 009-17 (Part I)
Site Master File (SMF) for Plasma Warehouses	PI 020-3
Site Master File (SMF) for Source Plasma Establishments	PI 019-3

Guidance for Inspectorates

The guidelines and references for the inspectorates are provided in detail in Table 3 [16].

**Table 3:** Inspectorate guidance

GUIDELINE	REFERENCE
Guidance on GMP Inspection Reliance	PI 048-1
Participating Authorities	PS/INF 21/2002 (Rev. 28)
PIC Convention of 1970	PIC Convention
PIC/S Audit Checklist	PS W 01 2005 (Rev. 3)
PIC/S Audit Checklist - Interpretation Guide	PS W 31 2019
PIC/S Guidelines for Accession	PS W 14 2011 (Rev. 3)
PIC/S Guidelines for the Pre-Accession Procedure	PS W 12 2019 (Rev. 1)
Procedure for Handling Rapid Alerts and Recalls Arising from Quality Defects	PI 010-5
Procedure on Notification of Foreign Inspections	PI 039-1
Quality System Requirements for Pharmaceutical Inspectorates	PI 002-3
Questionnaire for Assessment	PS W 01 2011 (Rev. 1)
Revised PIC/S Scheme	PICS 1/95 (Rev 6)
Standard Operating Procedure PIC/S Inspection Report Format	PI 013-3
Standard Operating Procedure Team Inspections	PI 031-1

Guidance for Inspectors

The guidelines and references for the inspectors are provided in detail in Table 4 [16].

**Table 4:** Inspector guidance

GUIDELINE	REFERENCE
Aide Memoire on Assessment of Quality Risk Management (QRM) Implementation	PI 038-2
Aide Memoire on GMP Particularities for Clinical Trial Products	PI 021-2
Aide Memoire on Inspection of Biotech	PI 024-3
Aide Memoire on Inspection of Quality Control Laboratories	PI 023-2
Aide Memoire on the Inspection of Health-Based Exposure Limit (HBEL) Assessments and Use in Quality Risk Management	PI 052-1
Aide-Memoire Inspection of Utilities	PI 009-4
Aide-Memoire on Cross-Contamination in Shared Facilities	PI 043-1
Aide-Memoire on Medicinal Gases	PI 025-2
Aide-Memoire on Packaging	PI 028-2
Aide-Memoire on the Inspection of Active Pharmaceutical Ingredients (APIs)	PI 030-1
Good Practices for Computerised Systems in Regulated GXP Environments	PI 011-3
Guidance on Classification of GMP Deficiencies	PI 040-1
Guidance on Parametric Release	PI 005-3
Guide to Good Practices for the Preparation of Medicinal Products in Healthcare Establishments	PE 010-4
Isolators Used for Aseptic Processing and Sterility Testing	PI 014-3
PIC/S Aide Memoire Inspection of GDP for Medicinal Products in the Supply Chain	PI 044-1
PIC/S Aide Memoire to Inspections of Blood Establishments and Plasma Warehouses	PI 008-4
PIC/S Good Practice Guidelines for Blood Establishments and Hospital Blood Banks	PE 005-4
PIC/S Good Practices for Data Management and Integrity in Regulated GMP/GDP Environments	PI 041-1
PIC/S Guidance COVID-19 Risk Assessment for Routine On-Site Inspections	PI 055-1
PIC/S Guide to Good Distribution Practice (GDP) for Medicinal Products	PE 011-1
PIC/S Guideline on Exposure Limits	PI 046-1
PIC/S Guidelines on Excipient GMP Risk Assessment	PI 045-1
PIC/S Guidelines on GDP of Active Substances for Human Use	PI 047-1
PIC/S Recommendation How to Evaluate and Demonstrate the Effectiveness of the Pharmaceutical Quality System With Regard to Risk-Based Change Management	PI 054-1
PIC/S Recommendation on Risk-Based Inspection Planning	PI 037-1
QA Distribution Activities for APIs - May 2010	PS INF 20 2011
QA on PIC/S GDP Guide (PE 011-1)	PS INF 22 2017
QA on Traceability of Medicinal Gases	PS INF 06 2012 (Rev 1)
Questions and Answers (Q&A) on the Implementation of Risk-Based Prevention of Cross-Contamination in Production and ‘Guideline on Setting Health-Based Exposure Limits (HBEL) for Use in Risk Identification in the Manufacture of Different Medicinal Products in Shared Facilities’	PI 053-1
Recommendation on Sterility Testing	PI 012-3
Recommendation on the Qualification and Training of Inspectors in the Field of Human Blood Tissues and Cells	PI 026-2
Validation Master Plan Installation and Operational Qualification Non-Sterile Process Validation Cleaning Validation	PI 006-3
Validation of Aseptic Processes	PI 007-6

Why compliance is important?

Possessing the necessary setups to implement an inspection system identical to the one referenced in this system, as well as the policies and procedures that could guarantee the Scheme's correct execution and support its efficient operation, is one of the fundamental prerequisites for competent authorities to join PIC/S. Being equal is always necessary, not just for admission, and it must be properly confirmed at reevaluations. Because of this, one of PIC/S's most crucial and significant areas that require ongoing supervision is compliance with the PIC scheme [3].

PIC/S sub-committee on compliance (SCC)

Since 2014, “SCC”, which is presently led by Mr. Henning Willads Petersen (Denmark / DKMA), has been in charge of overseeing compliance with the scheme.

Assessment and Pre-Assessment

Through the PIC/S's accession procedure, which may be preceded by a pre-accession process (for further information see "Accession"), PIC/S membership is typically attained after three to six years. A PIC/S delegation may need to conduct follow-up assessment visits to confirm the appropriateness of corrective actions if, during the assessment period, several adjustments and enhancements suggested by the PIC/S committee must be put into practice.

Re-Assessment

Only those who were evaluated for many years were applicants. Nevertheless, it never happened to evaluate the founding members. In 2000, a joint reassessment programme (JRP) was introduced to make sure that current PIC/S members (participating authorities) and new applicants met the same standards. As a result, current members are now routinely reevaluated for equivalency. To evaluate and reevaluate PIC/S members, there is just one PIC/S instrument available (Audit Checklist). Training should be provided to auditors using the JRP-JAP (Joint Audit Programme) training package as part of the PIC/S assessment and reassessment [17].

Strategic development

The founders of the PIC convention aimed to create a system that would both boost international trade and ensure exceptional patient safety through stringent GMP regulations. Their vision was to establish a free trade market for pharmaceutical products. To achieve this, they introduced the PIC convention, which facilitates mutual recognition of GMP inspections, and the PIC GMP Guide, which harmonizes GMP standards globally. However, in 1995, the convention's expansion faced a setback due to perceived legal conflicts with European regulations. In response, the PIC committee of officials developed a new strategy: a non-binding platform for sharing GMP-related information, advancing the harmonization of GMP standards, and providing GMP training, which resulted in the creation of the PIC plan.

This initiative involves developing and endorsing globally harmonized GMP guidelines and standards, educating competent authorities, particularly inspectors, evaluating and re-evaluating inspectorates and promoting collaboration and networking among competent authorities and global organizations.

In the coming years, the primary focus should be on assessing and enhancing the operational framework and structure of PIC/S. To improve operational effectiveness, it could be beneficial to implement more robust monitoring and evaluation mechanisms, increase transparency, and foster stronger collaboration with member authorities. It is crucial to ensure that strategic initiatives, such as those outlined in documents like the Blueprint, are effectively executed. Evaluating partnerships and identifying areas for improvement can further strengthen these relationships. Encouraging the active participation of governing bodies involved in the PIC scheme will also be advantageous. As stipulated in the terms of reference, providing the PIC/S committee with thoughtful suggestions and recommendations is essential for continuous improvement and alignment with the organization's objectives.

While the core goals and methods for achieving them remain relevant today, the future may present new challenges. Over the past 40 years, PIC/S has thrived due to its strategic and visionary approach. Moving forward, maintaining PIC/S's success will depend on ongoing strategic development.

PIC/S Working Groups Operating Under the Sub-committee on Strategic Development (SCSD)

Strategic development is being worked on by the PIC/S working groups which are working group on unique facility identifiers (UFI), working group on inspector travel safety, working group on informants, working group on inspection reliance, and working group on remote assessment [18].

Main benefits for members

PIC/S offers unique training opportunities for GMP inspectors through exclusive seminars, expert circles, and collaborative visits, setting it apart from other international platforms. By promoting the international harmonization of GMPs, PIC/S ensures a unified interpretation of GMP and quality systems, with members actively participating in the development and standardization of guidelines and recommendations. Networking opportunities at PIC/S events facilitate one-on-one interactions and information sharing among agencies, enhancing collaboration on GMP-related matters. The organization maintains high standards across all members, which bolsters the effectiveness of GMP inspection systems, particularly in training and quality system requirements. Additionally, members can share GMP inspection reports for active pharmaceutical ingredients (APIs), which streamlines inspection resources and reduces costs.

Indirect benefits for industry

As the industry's pertinent regulatory authority joins PIC/S, there are additional indirect advantages. The following are a few such advantages: Fewer inspections being duplicated, lower costs, better market access, and facilitation of exports. Although not a commerce accord, affiliation with PIC/S can boost drug exports. Some non-member agencies accept the quality compliance certificate of PIC/S members, indicating greater trust in medicines from these countries. Consequently, PIC/S affiliation significantly benefits their pharmaceutical industries [19].

PIC/S audit checklist

Summary of audit checklist - The audit checklist provides a thorough assessment of regulatory compliance and operational effectiveness across various areas. It starts with evaluating critical aspects such as empowering legislation and conflict of interest through documentation reviews and on-site inspections of the inspectorate. Regulatory directives are examined for procedures related to the appointment of inspectors, codes of conduct, and training policies, with some elements assessed in conjunction with other components. GMP standards are scrutinized for their detail and process validation.

Inspection resources, including staff qualifications, inspector numbers, and training programs, are reviewed through both documentation and on-site evaluations. Inspection procedures, such as strategy, preparation, reporting formats, and methodologies, are meticulously analyzed via documentation and observed inspections. Performance standards, enforcement authority, and alert systems are evaluated with a focus on managing non-compliance and crisis response mechanisms.

Analytical capabilities are assessed through documentation and laboratory inspections, covering aspects such as laboratory access, standard operating procedures (SOPs), and method validation. The surveillance program is reviewed for its sampling methods, audit procedures, and handling of consumer complaints. Lastly, the quality management system is examined through comprehensive documentation and on-site evaluations at both the inspectorate and laboratory [20].

## Conclusions

PIC and PIC schemes are now in synchronization as a result of PIC/S's implementation. It led to a shared understanding of GMP for medicines across the participating countries. PIC/S has provided drug regulatory bodies with a shared understanding of the introduction of consistent, high-quality, approved standard medicines that can be imported into member countries. There are 56 member authorities in PIC/S, and it operates under a well-defined framework comprising the PIC/S council, various working groups, an executive bureau, and a secretariat. Due to its robust and independent operational setup and legal foundation as a Swiss association, the scheme can readily adapt to evolving international standards.

The thorough evaluation of inspectorate and regulatory systems is emphasized through the extensive processes for new members' pre-accession and accession. PIC/S has benefited the pharmaceutical sector as a whole by saving time and money on drug approval processes, as well as governments, inspectors, and manufacturers. Joining PIC/S offers numerous advantages, including the opportunity to participate in exclusive training initiatives, the alignment of GMP regulations on a global scale, and enhanced networking opportunities. This leads to reduced expenses, decreased redundant inspections, and enhanced market entry for businesses. PIC/S strengthens the credibility and efficiency of pharmaceutical production worldwide and reinforces global regulatory frameworks through cooperation and standardization of protocols.
